# Dose–response relationship between transfusion and the incidence of infection in critically ill patients: a systematic review and dose–response meta-analysis

**DOI:** 10.1186/s40560-025-00822-x

**Published:** 2025-09-30

**Authors:** Shodai Yoshihiro, Yuki Kataoka, Takashi Hongo, Takahiro Tsuge, Hiroaki Matsuo

**Affiliations:** 1 Department of Pharmaceutical Services, Hiroshima University Hospital, Kasumi 1-2-3, Minami-ku, Hiroshima, 734-8551 Japan; 2 Scientific Research WorkS Peer Support Group (SRWS-PSG), Osaka, Japan; 3 Department of Internal Medicine, Kyoto Min-Iren Asukai Hospital, Tanaka Asukai-cho 89, Sakyo-ku, Kyoto, 606-8226 Japan; 4 Department of Healthcare Epidemiology, Graduate School of Medicine / Public Health, Kyoto University, Yoshida Konoe-cho, Sakyo-ku, Kyoto, 606-8501 Japan; 5 Department of Emergency, Critical Care, and Disaster Medicine, Dentistry, and Pharmaceutical Sciences, Okayama University Graduate School of Medicine, 2-5-1 Shikata-cho, Kita-ku, Okayama 700-8558 Japan; 6 Department of Rehabilitation, Kurashiki Medical Center, 250 Bakuro, Kurashiki, Okayama 710-8522 Japan; 7 Department of Epidemiology, Graduate School of Medicine, Dentistry and Pharmaceutical Sciences, Okayama University, 2-5-1 Shikata-cho, Okayama, 700-8558 Japan

**Keywords:** Critical care, Erythrocyte transfusion, Intensive care units, Sepsis

## Abstract

**Purpose:**

To estimate the association between red blood cell (RBC) transfusion volume and hospital-acquired infections (HAI) in critically ill patients, with a particular focus on identifying the potential threshold volumes at which infection risk changes.

**Methods:**

The MEDLINE, CENTRAL, Embase, and Transfusion Evidence Library databases were searched for studies published from database inception to November 2024. Citation searches and reference checks of the relevant guidelines were combined. Studies that evaluated transfusion and anemia pharmacotherapy in critically ill patients were included. Outcome of interest was the incidence of HAI. We conducted a dose–response meta-analysis (DRMA) using a one-stage random-effects model.

**Results:**

We identified 39,453 records after searching the databases. After combining the results of citation searches and reference checks of the guidelines, 45 studies were eligible. For the DRMA, we eliminated 14 studies without results and 15 with a critical risk of bias. We included 9587 patients from 16 studies. Our DRMA showed a non-linear risk curve, with odds ratio for HAI decreasing and reaching a trough at three units of RBC transfusion. Three units of RBC may not increase the risk of HAI in critically ill patients. However, the clinical implications of higher RBC transfusion volumes remain unclear.

**Conclusions:**

Our findings suggest a non-linear relationship between RBC transfusion volume and HAI risk in critically ill patients, highlighting the need for further research to inform individualized transfusion strategies.

*Clinical Trial Registration* http://osf.io/a9cwd

**Supplementary Information:**

The online version contains supplementary material available at 10.1186/s40560-025-00822-x.

## Introduction

Red blood cell (RBC) transfusion is one of the most frequently administered therapies in intensive care units (ICUs), with an administration rate of 25% during an ICU stay [1]. Red blood cell transfusions provide potential benefits by improving anemia and hemodynamics. This therapy carries inherent risks, including acute lung injury, circulatory overload, and hospital-acquired infections (HAI). Health-associated infections increase healthcare resource utilization and are associated with length of stay and economic burden [2]. Identification of modifiable risk factors is essential for optimizing patient outcomes.

The relationship between RBC transfusion and HAI in critically ill patients remains uncertain. Guideline-based systematic reviews (SR) focusing on randomized controlled trials (RCTs) published by major societies, including the French Intensive Care Society, European Society of Intensive Care Medicine (ESICM), and American College of Chest Physicians (CHEST), reported no difference in HAI risk between restrictive and liberal transfusion strategies for hemodynamically stable, non-bleeding patients [3–5]. However, guideline-based SRs primarily compared transfusion strategies based on hemoglobin thresholds, limiting their ability to evaluate the effect of actual transfusion volume and detect potential dose–response relationships. In contrast, observational studies suggested a potential incremental risk of infection with higher RBC transfusion volumes. A previous SR of observational studies demonstrated an association between the administration of ≥ 1 RBC units and increased HAI risk in critically ill patients [6]. Furthermore, one observational study reported that greater transfusion volume was independently associated with increased risk of HAI in trauma patients, demonstrating a clear dose–response trend [7]. This discrepancy suggests that infection risk is dose-dependent, raising the critical question: at what transfusion volume does the risk of HAI begin to increase, and can a safe threshold be identified?

To address this uncertainty, we conducted an SR and a dose–response meta-analysis (DRMA) to evaluate the relationship between RBC transfusion volume and HAI risk in critically ill patients.

## Methods

### Protocol and registration

A preplanned protocol for SR and DRMA has been registered in the Open Science Framework (Osf.io) [8]. The DRMA was conducted in accordance with the Preferred Reporting Items for Systematic Reviews and Meta-Analyses (PRISMA 2020) requirement [9]. The items mentioned in the PRISMA are shown in Supplementary Table 1.

### Eligibility criteria

Since we were interested in the association between the amount of transfusion and acquisition of infection in critically ill adult patients, we included randomized controlled trials (RCTs) and non-RCTs (NRCTs) that evaluated transfusion in critically ill patients. To evaluate dose–response relationships, there was a need for a difference in the amount of transfusion between the arms of these studies. The arms of such studies were ≥ 2 different amounts of transfusion within a study, including a no-transfusion group, where the amount of transfusion was 0 unit.

We included critically ill adult patients with hemodynamic stability who were admitted to the ICU. Elective surgery patients and those with burns were also excluded.

Outcome measure was the incidence of HAI. Healthcare-associated infections are bacterial, viral, and fungal infections acquired in hospitals where critically ill patients are admitted and newly developed apart from an underlying disease [10, 11]. We retrieved the number of patients with HAI during hospitalization. If HAI was not reported, ICU-acquired infections were recorded. We prioritized outcomes that were pre-defined as infections in the protocols of the primary studies. For the main analysis, we measured the category of infection using the following hierarchy: (i) sepsis/septic shock; (ii) bacteremia; and (iii) any infection recorded by original authors.

### Information sources

MEDLINE (PubMed), Cochrane Central Register of Controlled Trials (Cochrane Library), Embase (Dialog), and Transfusion Evidence Library (https://www.transfusionevidencelibrary.com) databases were searched for studies published from database inception to November 2024. ClinicalTrials.gov and the World Health Organization International Clinical Trials Platform Search Portal were also searched, and unpublished and ongoing studies were identified. Citations of eligible reports were manually searched using referenced articles and semantic scholars. Additionally, we searched studies included in guidelines for transfusion strategy [3, 4, 12]. We collected eligible studies from the search strategies for the network meta-analysis evaluating pharmacotherapies for critically ill patients and updated these search strategies till December 2024 [13]. Search terms were related to ICUs, brain injuries, and RBC/platelet transfusion. Detailed strategy used to search each database was described in our study protocol [8].

### Search strategy

After removing duplicate articles, we implemented a two-stage abstract screening using a hybrid approach combining human reviews and machine learning filters.

Three independent reviewers (SY, TH, and TT) manually screened 500 randomly selected records and resolved any disagreements through discussion. Using the reviewed records, we developed a first-stage machine-learning filter [14] with titles and abstracts as input features. To address dataset imbalance, we applied class weights.

From the records identified using the first filter, another manual screening of a 500-record random sample was performed. Based on this sample, we constructed a second-stage machine filter using the same methodology as in the first stage [15].

All machine learning was performed using Python (version 3.12.5; Python Software Foundation) with the following libraries: Scikit-learn (version 1.5.2), Optuna (version 4.1.0), and LightGBM (version 4.5.0). We used Bayesian optimization to determine the optimal hyperparameters to maximize the F4 score, which prioritizes sensitivity over specificity [15]. The F4 score was calculated as follows:

$$F4 = (1 + 4^{ \wedge } 2)\, \times (Specificity\, \times \,Sensitivity)/(4^{ \wedge } 2\, \times \,Specificity + Sensitivity)$$

Detailed scripts are available in the Github repository [16].

After adding the second filter, at least two independent reviewers (SY, TH, and TT) independently screened the titles and abstracts of the remaining articles. The same reviewers assessed the eligibility based on full texts. Throughout the screening process, disagreements between the two reviewers were resolved by discussion. If this failed, a third reviewer acted as an arbiter (YK).

### Evaluation of risk of bias

We applied the risk of bias 2.0 for RCTs and the risk of bias in non-randomized studies-of interventions version 2 (ROBINS-I V2) for NRCTs [17]. The risk of bias was independently assessed by at least two reviewers (SY, TH, and TT). YK mediated any unresolved disagreements. For reporting bias, we searched for unpublished trials in the trial registries.

### Data synthesis and generation of dose–response curves

A DRMA was conducted using a one-stage random-effects model implemented in the R package (version 4.4.2) dosresmeta (version 2.0.1; Maintained by Alessio Crippa) [18]. We pooled study-specific odds ratios (OR) with 95% confidence intervals (CI) to evaluate the association between the amount of transfusion and the risk of infection. To account for the potential nonlinearity of this relationship, we employed restricted cubic spline models. Specifically, we used three knots placed at the 25th, 50th, and 75th percentiles of the observed distribution of aggregated transfusion volumes across all studies. The variance–covariance matrix of the spline coefficients was estimated using maximum likelihood. Detailed scripts are available in the Github repository [19].

### Sensitivity analysis

We conducted sensitivity analyses by excluding studies that assessed pharmacotherapy for anemia in critically ill patients, NRCTs, follow-up periods (in-hospital stay and ICU stay), and critically ill patients with trauma.

### Important protocol deviation

We excluded studies with a critical risk of overall bias from the DRMA because many of the included studies were assessed as having a critical overall risk of bias. Because studies using imputed statistics were not included, a planned sensitivity analysis was not conducted. Additionally, we conducted sensitivity analyses divided into categories of HAI and a sensitivity analysis restricted to critically ill patients with trauma.

## Results

### Study selection

**Fig. 1:**
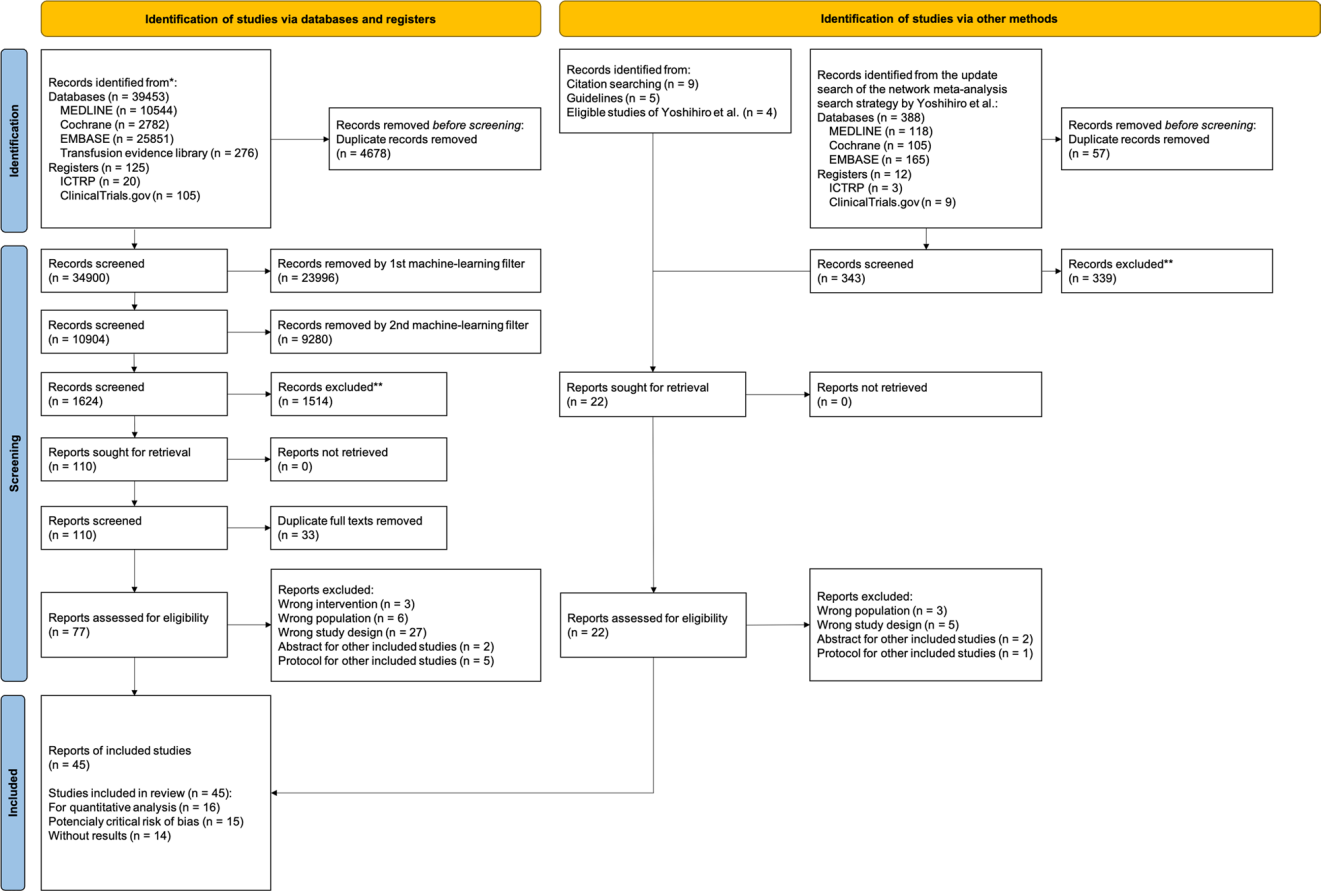
PRISMA flow diagram

A flow diagram of the study selection procedure is shown in Fig. 1. The database search identified 39,453 records. Among the initial 500 records manually screened, seven (1.4%) records met the inclusion criteria. The first filter achieved a sensitivity of 1.000 and a specificity of 0.704 in the training set. Applying the first filter to the remaining 34,900 records, 10,904 (31.2%) were selected for the next screening stage. In the second stage, manual screening of another random 500-record sample identified 27 (5.4%) relevant records. The second filter demonstrated a sensitivity of 1.000 and a specificity of 0.896. Applying the second filter to the 10,904 records, 1624 (14.9%) records were retained for full reviews. Following a human review, we included 34 studies. We included eight additional studies: four from the reference lists of a network meta-analysis of pharmacotherapies for critically ill patients [13], and four through an updated literature search. After combining the results from the citation searches and reference checks of the relevant guidelines, we included three studies in the meta-analysis. Ultimately, 45 studies were included in the analysis. After full-text screening of the 99 reports, the reasons for exclusion are presented in Supplementary Table 2.

### Study characteristics

Of the 45 studies, 14 without results were included; details are presented in Supplementary Table 3 [7, 20–32]. The included RCTs compared transfusion strategies [33–38], pharmacotherapies for anemia [39–42], and the storage period of RBC [43]. The included NRCTs evaluated the association between transfusion volume and HAI in critically ill patients by categorizing them into two groups based on the presence or absence of transfusion use [44–49], occurrence of HAI [50–58], and others [59–63].

Of 31 studies, 15 NRCTs that were categorized into two groups based on the presence or absence of transfusion use and the occurrence of HAI were excluded from DRMA due to the critical immortal time bias; a methodological error where the period between cohort entry and treatment initiation is misclassified as “event-free” survival time, potentially leading to overestimation of treatment benefits. Details of the 15 NRCTs are presented in Supplementary Table 4. As the NRCTs have no pre-registered protocols, the overall risk of bias was moderate. There were some concerns regarding the randomization process in six RCTs. The fact that the measurement of HAI was not described in the protocol was also a cause of bias. The risk of bias is presented in Supplementary Figs. 1–4. No reporting bias was observed.

The DRMA comprised 16 studies, including 11 RCTs and five NRCTs. Of 9587 patients, 5812 were transfused with RBC; although the number of transfused patients was not obtained from one study [62]. Table 1 shows patient characteristics. As platelet volume was reported in only two studies [40, 43], DRMA was not conducted.

**Table 1:** Characteristics of studies for quantitative analysis

Author year	Design	Nature of participants	Sample size	Age, years	Hemo-globin, g/dL	Gender, male, %	Comparison type	Comparison1: hemoglobin trigger/Target (g/dL)	Comparison2: hemoglobin trigger/Target (g/dL)
Corwin [39]	RCT	Critical illness	1302	51	10	62	Pharmacological	< 9 (or hematocrit < 27%)/NA	None
Corwin [41]	RCT	Critical illness	1448	50.3	9.6	63.1	Pharmacological	NA/7–9	None
Earley [59]	NRCT	Trauma	514	43.3	NA	71.6	Management Program	NA/7–9	None
English [36]	RCT	SAH	732	59.4	9.4	18.3	Transfusion Strategy	≤ 8/NA	≤ 10/NA
Fernandez [60]	NRCT	Critical illness	62	63	12.7	67.7	Biomarker-guided	< 8/NA	None
Georgopoulos [40]	RCT	Critical illness	99	60.3	9.3	76.4	Pharmacological	NA/7–9	None
Gobatto [35]	RCT	Trauma	44	34.6	8.8	90.8	Transfusion Strategy	< 7/NA	< 9/NA
Hebert [33]	RCT	Critical illness	838	57.6	9.6	62.5	Transfusion Strategy	NA/7–9	NA/10–12
Michetti [61]	NRCT	Trauma	1038	54.6	NA	70.3	Order Set	< 7/NA	Unclear
Pieracci [42]	RCT	Trauma	150	41	NA	69.3	Pharmacological	NA/7–9	None
Robertson [34]	RCT	Trauma	200	30.1	14.4	87	Transfusion Strategy	≤ 7/NA	≤ 10/NA
Schreiber [43]	RCT	Trauma	167	49.8	NA	39.8	RBC Type/Storage	Unclear	Unclear
Taccone [37]	RCT	Trauma	820	51.5	8.5	54.1	Transfusion Strategy	< 7/NA	< 9/NA
Turgeon [38]	RCT	Trauma	736	48.7	9.8	72.7	Transfusion Strategy	≤ 7/NA	≤ 10/NA
Xiao [63]	NRCT	Trauma	1252	42.6	6.8	69	RBC Age	≤ 8/Close to 10	None
Yahia [62]	NRCT	Critical illness	50	62.6	6.5	52	Transfusion Strategy	≤ 7/7.1–9	≤ 9/9.1–11

*EPA* erythropoietin, *ICU* intensive care unit, *NA* not applicable, *NRCT* non-randomized controlled trial, *RBC* red blood cell, *RCT* randomized controlled trial, *SAH* subarachnoid hemorrhage, *VAP* ventilator-associated pneumonia

Hemoglobin Trigger/Target is value for hemodynamically stable critically ill patients

### Hospital acquired infections

**Fig. 2:**
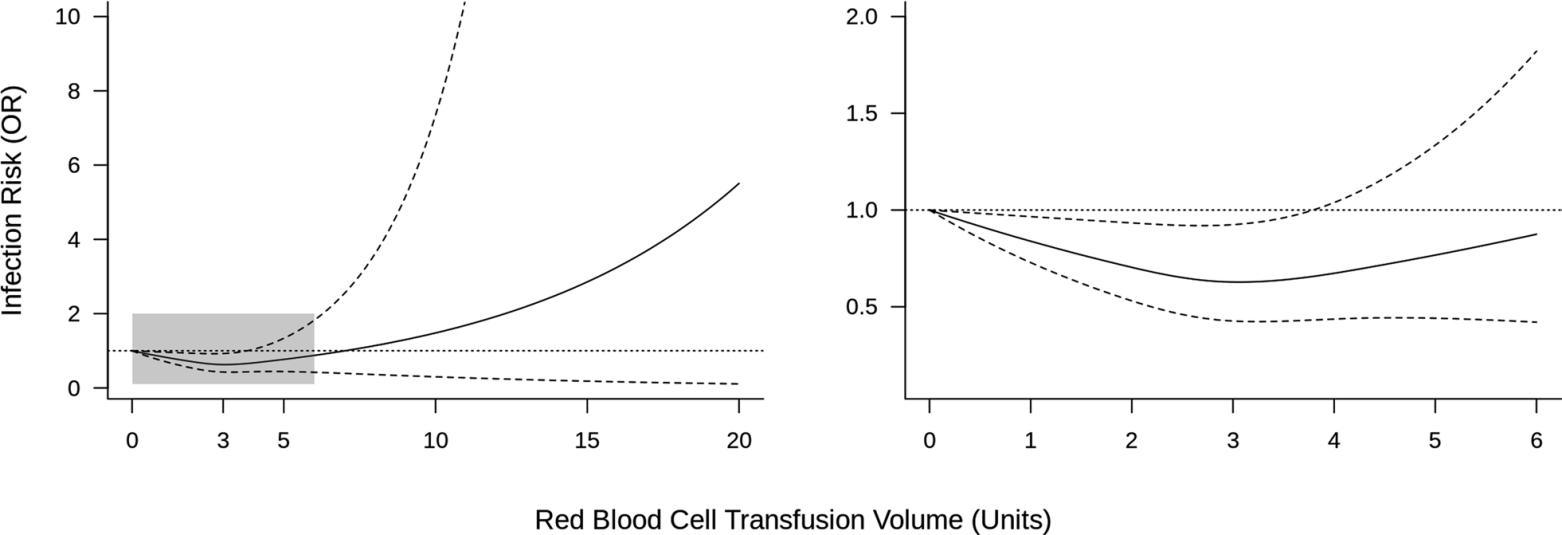
Dose analysis curve between RBC transfusion volume and the incidence of HAI. The graph illustrates the odds ratio (OR) for healthcare-associated infections (HAI) in relation to red blood cell (RBC) transfusion volume (units). The solid line represents the adjusted risk estimate and the dashed lines indicate 95% confidence intervals. The dotted horizontal line at OR = 1 represents no change in the infection risk. The shaded area in the left panel indicates the magnified region in the right panel. In the right panel, a magnified view of the 0–6 units range shows that the infection risk has reached its minimum at approximately three units, after which the risk begins to increase.

A dose-dependent relationship between HAI and RBC transfusion volume was determined in 16 studies involving 9587 patients (Fig. 2). In 9587 patients, 700 patients (7.3%) developed HAI. Seven studies measured HAI only during ICU stay. Applying 0 unit of RBC transfusion as a reference, the OR for HAI may decrease from 0.63 (95% CI 0.43–0.92) at three units (Supplementary Table 5). Although the point estimates shifted from 0.77 (0.44–1.34) at five units to 1.48 (0.30–7.34) at ten units, it was uncertain (Supplementary Table 5). The sensitivity analysis results supported the robustness and reliability of the primary analysis at approximately three units (Supplementary Figs. 5–13).

Sepsis/septic shock was reported in nine studies involving 6373 patients, bacteremia in five studies involving 2550 patients, any infection in nine studies involving 3475 patients, and pneumonia in nine studies involving 5668 patients. The dose-dependent relationships of these analyses are presented in Supplementary Figs. 9–12. The subgroup analyses by type of HAI showed point estimates that followed a similar trend to the primary outcome, with the lowest estimated risk occurring around three units of RBC transfusion (Supplementary Figs. 9–12). Point estimates for RBC transfusions of three or more units were in the opposite direction for sepsis/septic shock and other categories.

## Discussion

We conducted a DRMA to estimate the association between RBC transfusion volume and HAI acquisition in critically ill patients. Our DRMA included 16 studies and involved 9587 patients, excluding NRCTs with a critical risk of bias. The analysis revealed a non-linear U-shaped risk curve, with the odds of HAI decreasing and reaching a minimum at approximately three units of RBC transfusion. Beyond this threshold, the risk trajectory became uncertain owing to limited data and potential heterogeneity among studies. These findings suggest that transfusion of up to three units may not increase the risk of HAI in critically ill patients. However, the effects of high transfusion volumes remain unclear and require further investigation.

Our findings provide novel insights into the relationship between transfusion volume and HAI risk, suggesting that the risk may not increase up to approximately three units of RBC transfusion. While our analysis primarily included observational studies, we addressed potential biases by excluding studies with critical risk of bias and conducting sensitivity analyses. These efforts reduced heterogeneity, especially among studies with lower transfusion volumes, strengthening the robustness of our findings within that low exposure range. Transfusions may increase HAI risk in critically ill patients on the previous SR; however, that conclusion was largely based on studies with a critical risk of bias [6]. Our results indicate that, at least within the current clinical practice of restrictive transfusion strategies recommended by guidelines [3–5], RBC transfusion may not necessarily increase the risk of HAI. These findings suggest that healthcare professionals should incorporate transfusion volume into individualized HAI management for critically ill patients.

Molecular mechanisms may support the existence of the inflection point at which RBC transfusion dose alters the risk of HAI. Red blood cell metabolism affects macrophage polarization, shifting from a pro-inflammatory (M1) to an anti-inflammatory (M2) phenotype, which in turn modulates immune responses [64]. This phenotypic shift was related to the metabolic rate of RBCs phagocytosed by macrophages [64]. Type M2 macrophages secrete interleukin-10 and transforming growth factor-β during RBC metabolism and promote an anti-inflammatory action and suppress excessive immune responses [64–66]. Upon exposure to three units of erythrocyte transfusion, the systemic inflammatory response associated with sepsis might decrease as a consequence of increased M2 macrophage activity, and the OR of HAI may gradually decrease, as shown in our main analysis.

Further studies are required to determine the association between RBC transfusion volume and HAI acquisition in critically ill patients. In our sensitivity analysis, exposure to > 3 units of RBC transfusion was associated with a decreased risk of sepsis/septic shock, whereas an increased risk was observed in other categories. These results support the potential for immunosuppressive effects when RBC transfusions exceeded three units, resulting in a reduction in sepsis/septic shock and an increased risk of other HAI. Notably, these findings regarding high-volume transfusions were primarily derived from NRCTs that lacked time-dependent modeling. To clarify the dose–response relationship and minimize bias, future studies should adopt more sophisticated analytical approaches that account for both potential confounding factors and the time-varying nature of transfusion exposure and follow-up time.

Our SR and DRMA methods have some limitations. First, we developed the machine learning model to assist in study selection, although such a model could not completely eliminate the oversight. To minimize false negatives, the model was optimized accordingly, and we conducted a manual citation search to ensure comprehensive inclusion of relevant studies. Second, we could not conduct a DRMA to estimate the association between the platelet transfusion volume and HAI acquisition in critically ill patients. A continuous estimation is needed to clarify the effect of platelet transfusion volume on individual HAI. Third, in this DRMA, we included NRCT to obtain a sufficient amount of evidence. Since RBC exposure is likely associated with survival time, our analysis may not eliminate immortal time bias between patients in each volume of RBC transfusion. To ensure the quality of evidence, we excluded studies with a critical risk bias as assessed by ROBINS-I. Fourth, the proportion of HAI in this DRMA is smaller than that in the epidemiologic study [67], which may underestimate the impact of RBC transfusion. Finally, we could not restrict the categories of HAI and follow-up duration in the eligible studies, and patient backgrounds were not perfectly matched. As a result, we could not explore more detailed heterogeneity with the available dataset. Especially, in this analysis, many of the included studies focused on trauma patients, and therefore the results might limit generalizability to all critically ill patients. However, among the included patients, hematological diseases were present in only 45/9587 (0.45%) and cardiac diseases in 574/9587 (6.0%). Given these very small proportions, we consider it unlikely that they significantly influenced the results. Although sensitivity analyses show the robustness of results in the low-dose range, further research is needed to clarify the sources and impact of heterogeneity.

## Conclusion

In our DRMA, < 3 units of RBC did not increase HAI in critically ill patients. However, the association between RBC transfusions of ≥ 3 units and the effect on HAI in critically ill patients is uncertain.

## Supplementary Information

Additional file 1.

## Data Availability

The datasets analyzed during the current study are available in the Github repository [19].

## Abbreviations

DRMA: Dose–response meta-analysis
EPA: Erythropoietin
HAI: Hospital-acquired infections
ICU: Intensive care unit
NA: Not applicable
NRCT: Non-randomized controlled trial
OR: Odds ratios
RBC: Red blood cell
RCT: Randomized controlled trial
SAH: Subarachnoid hemorrhage
VAP: Ventilator-associated pneumonia
