# Supplementary material for: Dose–response relationship between transfusion and the incidence of infection in critically ill patients: a systematic review and dose–response meta-analysis
Source: J Intensive Care. 2025 Sep 30;13:53. doi: 10.1186/s40560-025-00822-x (PMC12486664; doi:10.1186/s40560-025-00822-x)
Supplement: Supplementary file 1 — Additional file 1. [file 40560_2025_822_MOESM1_ESM.docx]

**The dose-response relationship between transfusion and the incidence of infection in critically ill patients: A systematic review and dose-response meta-analysis.**

**Supplementary file**

Contents

[Table S1. PRISMA checklist 2020. 2](#_Toc202563343)

[Table S2. Excluded records by full-text screening. 5](#_Toc202563344)

[Table S3. Studied without results. 7](#_Toc202563345)

[Table S4. Non-randomized controlled studies with critical risk of bias. 8](#_Toc202563346)

[Table S5. Odds Ratios for HAI by RBC Transfusion Volume. 9](#_Toc202563347)

[Figure S1. Traffic light plots for randomized controlled trials. 10](#_Toc202563348)

[Figure S2. Traffic light plots for non-randomized controlled trials. 11](#_Toc202563349)

[Figure S3. Bar plots for randomized controlled trials. 12](#_Toc202563350)

[Figure S4. Bar plots for non-randomized controlled trials. 12](#_Toc202563351)

[Figure S5. Sensitivity analysis excluding studies to assess the pharmacotherapy for anemia. 13](#_Toc202563352)

[Figure S6. Sensitivity analysis excluding non-randomized controlled trials. 13](#_Toc202563353)

[Figure S7. Sensitivity analysis for studies measuring ICU-acquired infections. 14](#_Toc202563354)

[Figure S8. Sensitivity analysis for studies measuring Hospital-acquired infections. 14](#_Toc202563355)

[Figure S9. Sensitivity analysis for hospital-acquired infections in patients with sepsis/septic shock. 15](#_Toc202563356)

[Figure S10. Sensitivity analysis for hospital-acquired infections in patients with bacteremia. 15](#_Toc202563357)

[Figure S11. Sensitivity analysis for hospital-acquired infections in patients with any infections. 16](#_Toc202563358)

[Figure S12. Sensitivity analysis for hospital-acquired infections in patients with pneumonia. 16](#_Toc202563359)

[Figure S13. Sensitivity analysis for critically ill patients with trauma. 17](#_Toc202563360)

[e-References 18](#_Toc202563361)

# **Table S1. PRISMA checklist 2020.**

| **Section and Topic** | **Item #** | **Checklist item** | **Location where item is reported** |
| --- | --- | --- | --- |
| **TITLE** | | |  |
| Title | 1 | Identify the report as a systematic review. | Page 1 |
| **ABSTRACT** | | |  |
| Abstract | 2 | See the PRISMA 2020 for Abstracts checklist. | Page 2 |
| **INTRODUCTION** | | |  |
| Rationale | 3 | Describe the rationale for the review in the context of existing knowledge. | Page 4 |
| Objectives | 4 | Provide an explicit statement of the objective(s) or question(s) the review addresses. | Page 4 |
| **METHODS** | | |  |
| Eligibility criteria | 5 | Specify the inclusion and exclusion criteria for the review and how studies were grouped for the syntheses. | Page 5 |
| Information sources | 6 | Specify all databases, registers, websites, organisations, reference lists and other sources searched or consulted to identify studies. Specify the date when each source was last searched or consulted. | Page 5, 6 |
| Search strategy | 7 | Present the full search strategies for all databases, registers and websites, including any filters and limits used. | Protocol (Reference #7) |
| Selection process | 8 | Specify the methods used to decide whether a study met the inclusion criteria of the review, including how many reviewers screened each record and each report retrieved, whether they worked independently, and if applicable, details of automation tools used in the process. | Page 6, 7 |
| Data collection process | 9 | Specify the methods used to collect data from reports, including how many reviewers collected data from each report, whether they worked independently, any processes for obtaining or confirming data from study investigators, and if applicable, details of automation tools used in the process. | Protocol (Reference #7) |
| Data items | 10a | List and define all outcomes for which data were sought. Specify whether all results that were compatible with each outcome domain in each study were sought (e.g. for all measures, time points, analyses), and if not, the methods used to decide which results to collect. | Page 5 |
|  | 10b | List and define all other variables for which data were sought (e.g. participant and intervention characteristics, funding sources). Describe any assumptions made about any missing or unclear information. | Protocol (Reference #7) |
| Study risk of bias assessment | 11 | Specify the methods used to assess risk of bias in the included studies, including details of the tool(s) used, how many reviewers assessed each study and whether they worked independently, and if applicable, details of automation tools used in the process. | Page 7 |
| Effect measures | 12 | Specify for each outcome the effect measure(s) (e.g. risk ratio, mean difference) used in the synthesis or presentation of results. | Page 7 |
| Synthesis methods | 13a | Describe the processes used to decide which studies were eligible for each synthesis (e.g. tabulating the study intervention characteristics and comparing against the planned groups for each synthesis (item #5)). | Page 7 |
|  | 13b | Describe any methods required to prepare the data for presentation or synthesis, such as handling of missing summary statistics, or data conversions. | Protocol (Reference #7) |
|  | 13c | Describe any methods used to tabulate or visually display results of individual studies and syntheses. | Page 7 |
|  | 13d | Describe any methods used to synthesize results and provide a rationale for the choice(s). If meta-analysis was performed, describe the model(s), method(s) to identify the presence and extent of statistical heterogeneity, and software package(s) used. | Page 7 |
|  | 13e | Describe any methods used to explore possible causes of heterogeneity among study results (e.g. subgroup analysis, meta-regression). | Page 7 |
|  | 13f | Describe any sensitivity analyses conducted to assess robustness of the synthesized results. | Page 7 |
| Reporting bias assessment | 14 | Describe any methods used to assess risk of bias due to missing results in a synthesis (arising from reporting biases). | Page 7 |
| Certainty assessment | 15 | Describe any methods used to assess certainty (or confidence) in the body of evidence for an outcome. | Not appricable |
| **RESULTS** | | |  |
| Study selection | 16a | Describe the results of the search and selection process, from the number of records identified in the search to the number of studies included in the review, ideally using a flow diagram. | Page 8 |
|  | 16b | Cite studies that might appear to meet the inclusion criteria, but which were excluded, and explain why they were excluded. | Page 8, Supple-mentary Table 2 |
| Study characteristics | 17 | Cite each included study and present its characteristics. | Page 8, Table 1 |
| Risk of bias in studies | 18 | Present assessments of risk of bias for each included study. | Page 9,  Supple-mentary Figure 1-4 |
| Results of individual studies | 19 | For all outcomes, present, for each study: (a) summary statistics for each group (where appropriate) and (b) an effect estimate and its precision (e.g. confidence/credible interval), ideally using structured tables or plots. | Page 9,  Figure 2 |
| Results of syntheses | 20a | For each synthesis, briefly summarise the characteristics and risk of bias among contributing studies. | Page 9 |
|  | 20b | Present results of all statistical syntheses conducted. If meta-analysis was done, present for each the summary estimate and its precision (e.g. confidence/credible interval) and measures of statistical heterogeneity. If comparing groups, describe the direction of the effect. | Page 9 |
|  | 20c | Present results of all investigations of possible causes of heterogeneity among study results. | Page 9, 10, Supple-mentary Figure 5-13 |
|  | 20d | Present results of all sensitivity analyses conducted to assess the robustness of the synthesized results. | Page 9, 10, Supple-mentary Figure 5-13 |
| Reporting biases | 21 | Present assessments of risk of bias due to missing results (arising from reporting biases) for each synthesis assessed. | Page 9,  Supple-mentary Table 3 |
| Certainty of evidence | 22 | Present assessments of certainty (or confidence) in the body of evidence for each outcome assessed. | Not appricable |
| **DISCUSSION** | | |  |
| Discussion | 23a | Provide a general interpretation of the results in the context of other evidence. | Page 10 |
|  | 23b | Discuss any limitations of the evidence included in the review. | Page 11 |
|  | 23c | Discuss any limitations of the review processes used. | Page 11 |
|  | 23d | Discuss implications of the results for practice, policy, and future research. | Page 11 |
| **OTHER INFORMATION** | | |  |
| Registration and protocol | 24a | Provide registration information for the review, including register name and registration number, or state that the review was not registered. | Reference #7 |
|  | 24b | Indicate where the review protocol can be accessed, or state that a protocol was not prepared. | Reference #7 |
|  | 24c | Describe and explain any amendments to information provided at registration or in the protocol. | Page 7-8 |
| Support | 25 | Describe sources of financial or non-financial support for the review, and the role of the funders or sponsors in the review. | Page 12 |
| Competing interests | 26 | Declare any competing interests of review authors. | Page 12 |
| Availability of data, code and other materials | 27 | Report which of the following are publicly available and where they can be found: template data collection forms; data extracted from included studies; data used for all analyses; analytic code; any other materials used in the review. | Page 7 |

From: Page MJ, McKenzie JE, Bossuyt PM, Boutron I, Hoffmann TC, Mulrow CD, et al. The PRISMA 2020 statement: an updated guideline for reporting systematic reviews. BMJ 2021;372:n71. doi: 10.1136/bmj.n71. This work is licensed under CC BY 4.0. To view a copy of this license, visit <https://creativecommons.org/licenses/by/4.0/>

# **Table S2. Excluded records by full-text screening.**

| Study_ID | Reasons for exclusion | The details |
| --- | --- | --- |
| Aubron 2017 | Wrong study design | No information for transfusion volume and/or infection |
| Audibert 2014 | Wrong study design | No information for transfusion volume and/or infection |
| Bergamin 2014 | Wrong population |  |
| Borges 2019 | Wrong intervention |  |
| Carson 2021 | Wrong population |  |
| Carson 2023 MINT | Wrong population | There is no obvious reference to patients admitted to the ICU in the publication |
| Chantepie 2015 | Wrong population |  |
| Cooper 2011 | Wrong population | There is no obvious reference to patients admitted to the ICU in the publication |
| Elsheikh 2024 | Wrong study design | No information for transfusion volume and/or infection |
| Fenwick 1994 | Wrong study design |  |
| Fujita 2014 | Wrong study design |  |
| Gobatto 2017 Gobatto 2019 | Abstract for other included studies |  |
| Gobatto 2019 registry | Protocol for other included studies |  |
| Hagerty 2015 | Wrong study design | No information for transfusion volume and/or infection |
| Hayakawa 2023 | Wrong study design |  |
| Hebert 2001 | Wrong study design | No information for transfusion volume and/or infection |
| Holst 2014 protocol | Protocol for other included studies |  |
| Jairath 2015 | Wrong population | There is no obvious reference to patients admitted to the ICU in the publication |
| Kaukonen 2014 protocol | Wrong study design | Same amount of transfusion in each group |
| Koseoglu 2023 | Wrong study design |  |
| Kramer 2008 | Wrong study design | Same amount of transfusion in each group |
| Lacroix 2015 | Wrong study design | Same amount of transfusion in each group |
| Leger 2023 | Wrong study design | No information for transfusion volume and/or infection |
| Lelubre 2016 | Wrong study design |  |
| Levine 2010 | Wrong study design | No information for transfusion volume and/or infection |
| Loudon 2019 | Wrong study design | No information for transfusion volume and/or infection |
| Mamak 2011 | Wrong study design | No information for transfusion volume and/or infection |
| Matthew 2019 | Wrong study design | No information for transfusion volume and/or infection |
| McIntyre 2006 | Wrong study design | Same amount of transfusion in each group |
| Muller 2015 protocol | Wrong study design | Same amount of transfusion in each group |
| Mullis 2024 | Wrong population |  |
| Mustahsin 2023 | Wrong intervention |  |
| Naidech 2010 | Wrong study design | Same amount of transfusion in each group |
| Naidech 2010 | Abstract for other included studies |  |
| NCT06670963 2024 02 | Protocol for other included studies |  |
| Nilsson 2020 | Wrong study design | No information for transfusion volume and/or infection |
| Noval 1999 | Wrong study design |  |
| Parsons 2011 | Wrong study design | No information for transfusion volume and/or infection |
| Pedrosa 2021 | Wrong study design | No information for transfusion volume and/or infection |
| Peju 2019 Peju 2021 | Abstract for other included studies |  |
| Roberts 2012 | Wrong study design |  |
| Russell 2018 Holst 2014 | Wrong study design | No information for transfusion volume and/or infection |
| Sathe 2021 | Wrong population |  |
| Siegal 2023 a | Wrong study design | No information for transfusion volume and/or infection |
| Siegal 2023 b | Wrong study design | No information for transfusion volume and/or infection |
| Siegal 2023 registry | Wrong study design | No information for transfusion volume and/or infection |
| Suzuki 2022 | Wrong intervention |  |
| Taccone 2024 protocol | Protocol for other included studies |  |
| Turgeon 2022 registry | Protocol for other included studies |  |
| Turgeon 2022 Turgeon 2024 protocol | Protocol for other included studies |  |
| Villanueva 2013 | Wrong population | There is no obvious reference to patients admitted to the ICU in the publication |
| Wang 2021 | Abstract for other included studies |  |
| Yaseen 2023 | Wrong study design |  |
| Younan 2020 | Wrong study design | No information for transfusion volume and/or infection |

ICU, intensive care units.

# **Table S3. Studied without results.**

| Author Year | Nature of participants | Interventions | Comparisons | Recruitment status |
| --- | --- | --- | --- | --- |
| Aubron 2012 [1] | Critical illness | Short storage red cell transfusion | Standard care |  |
| Bergamin 2017 [2] | Critical illness | Restrictive transfusion strategy | Liberal transfusion strategy |  |
| Fogagnolo 2024 [3] | Critical illness | Oxygen extraction ratio-guided transfusion strategy | Hemoglobin-guided transfusion strategy | Ongoing |
| Hebert 1995 [4] | Critical illness | Restrictive transfusion strategy | Liberal transfusion strategy |  |
| Holst 2014 [5] | Sepsis | Restrictive transfusion strategy | Liberal transfusion strategy |  |
| Juffermans 2011 [6] | Sepsis | No-transfusion | Transfusion |  |
| Leal-Noval 2016 [7] | Critical illness | Regional cerebral oxygen saturation-  guided transfusion strategy | Hemoglobin-guided transfusion strategy |  |
| NCT03837171 2019 [8] | Critical illness | Restrictive transfusion strategy | Liberal transfusion strategy | No publication |
| NCT06670963 2024 [9] | Sepsis | Epoetin alfa plus iron | Normal saline | Ongoing |
| Nederpelt 2020 [10] | Trauma | Infection | no-infection |  |
| Walsh 2004 [11] | Critical illness | Fresh RBC transfusion | Old RBC transfusion |  |
| Walsh 2013 [12] | Critical illness | Restrictive transfusion strategy | Liberal transfusion strategy |  |
| Wang 2022 [13] | Critical illness | VAP | no-VAP |  |
| Zhang 2019 [14] | Trauma | Low-dose, early fresh frozen plasma transfusion | Normal saline |  |

RBC, red blood cell; VAP, ventilator-associated pneumonia.

# **Table S4. Non-randomized controlled studies with critical risk of bias.**

| Author Year | Design | Nature of participants | Sample size | Age, years | Hemoglobin, g/dL | Gender, male, % | Interventions | Control |
| --- | --- | --- | --- | --- | --- | --- | --- | --- |
| Bochicchio 2008 [15] | NRCT | Trauma | 766 | 42.4 | NA | 74.2 | VAP | No-VAP |
| Dupuis 2017 [16] | NRCT | Sepsis | 6016 | 65.3 | NA | 62 | Transfusion | No-transfusion |
| Engele 2016 [17] | NRCT | Critical illness | 3502 | 59.1 | NA | 60.7 | Infected | Non-infected |
| George 2008 [18] | NRCT | Trauma | 82 | 53.6 | 8.9 | 65.9 | Transfusion | No-transfusion |
| Juffermans 2012 [19] | NRCT | Trauma | 196 | 39.9 | NA | 76.1 | Infected | Non-infected |
| Leal-Noval 2013 [20] | NRCT | Critical illness | 428 | 63 | 9.7 | 60 | Transfusion | No-transfusion |
| Michalia 2012 [21] | NRCT | Critical illness | 330 | 56.2 | NA | 75.8 | Blood stream infection | No-blood stream infection |
| Offner 2002 [22] | NRCT | Trauma | 61 | 37.6 | NA | 80.3 | Infected | Non-infected |
| Peju 2021 [23] | NRCT | Sepsis | 893 | 68.4 | 7.6 | 63.5 | ICU-acquired infection | No-ICU-acquired infection |
| Piriyapatsom 2014 [24] | NRCT | Critical illness | 288 | 65.7 | 10.4 | 52.8 | Transfusion | No-transfusion |
| Rachoin 2009 [25] | NRCT | Critical illness | 2432 | 61.5 | NA | 52.6 | Transfusion | No-transfusion |
| Shorr 2005 [26] | NRCT | Critical illness | 3502 | 60.2 | NA | 54.9 | Blood stream infection | No-blood stream infection |
| Taylor 2002 [27] | NRCT | Critical illness | 1717 | NA | NA | NA | Infected | Non-infected |
| Taylor 2006 [28] | NRCT | Critical illness | 2085 | 59.8 | NA | 52.7 | Transfusion | No-transfusion |
| Zilberberg 2008 [29] | NRCT | Critical illness | 4344 | 61.5 | 11.7 | 54.5 | Transfusion | No-transfusion |

ICU, intensive care units; NA, not applicable NRCT, non-randomized controlled trials; VAP, ventilator-associated pneumonia.

# **Table S5. Odds Ratios for HAI by RBC Transfusion Volume.**

| Unit of RBC transfusion | Odds ratio (95% confidence intervals) |
| --- | --- |
| 1 | 0.84 (0.73–0.97) |
| 2 | 0.70 (0.53–0.93) |
| 3 | 0.63 (0.43–0.92) |
| 4 | 0.67 (0.44–1.04) |
| 5 | 0.77 (0.44–1.34) |
| 6 | 0.88 (0.42–1.82) |
| 7 | 1.00 (0.39–2.54) |
| 8 | 1.14 (0.36–3.60) |
| 9 | 1.30 (0.33–5.13) |
| 10 | 1.48 (0.30–7.34) |

The group receiving 0 units was used as the reference category.

# **Figure S1. Traffic light plots for randomized controlled trials.**


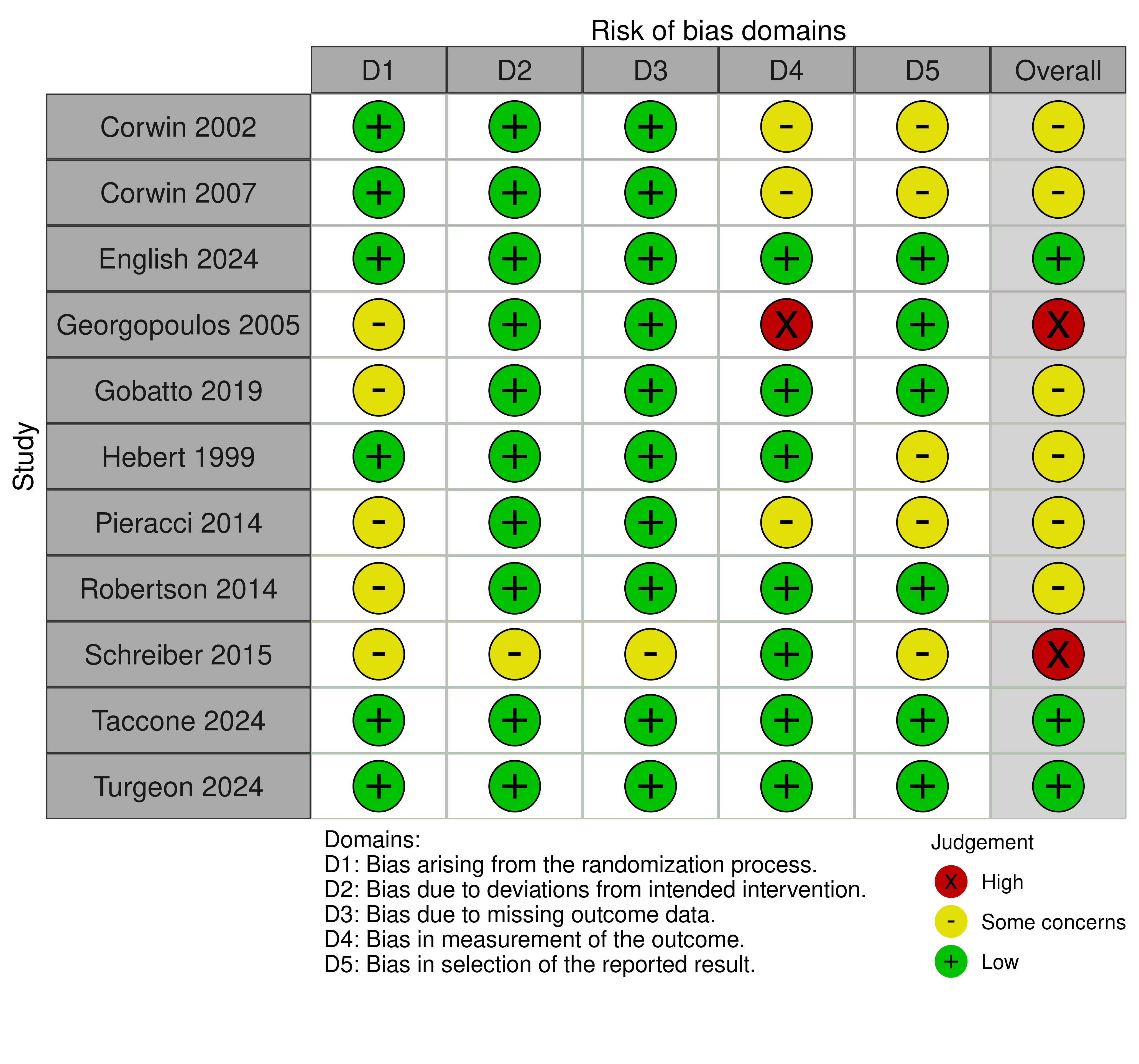


# **Figure S2. Traffic light plots for non-randomized controlled trials.**

We assessed the risk of bias by using ROVIS-I version 2 in non-randomized controlled trials, and domain 1 was rated as “low expect for concerns about uncontrolled confounding”.


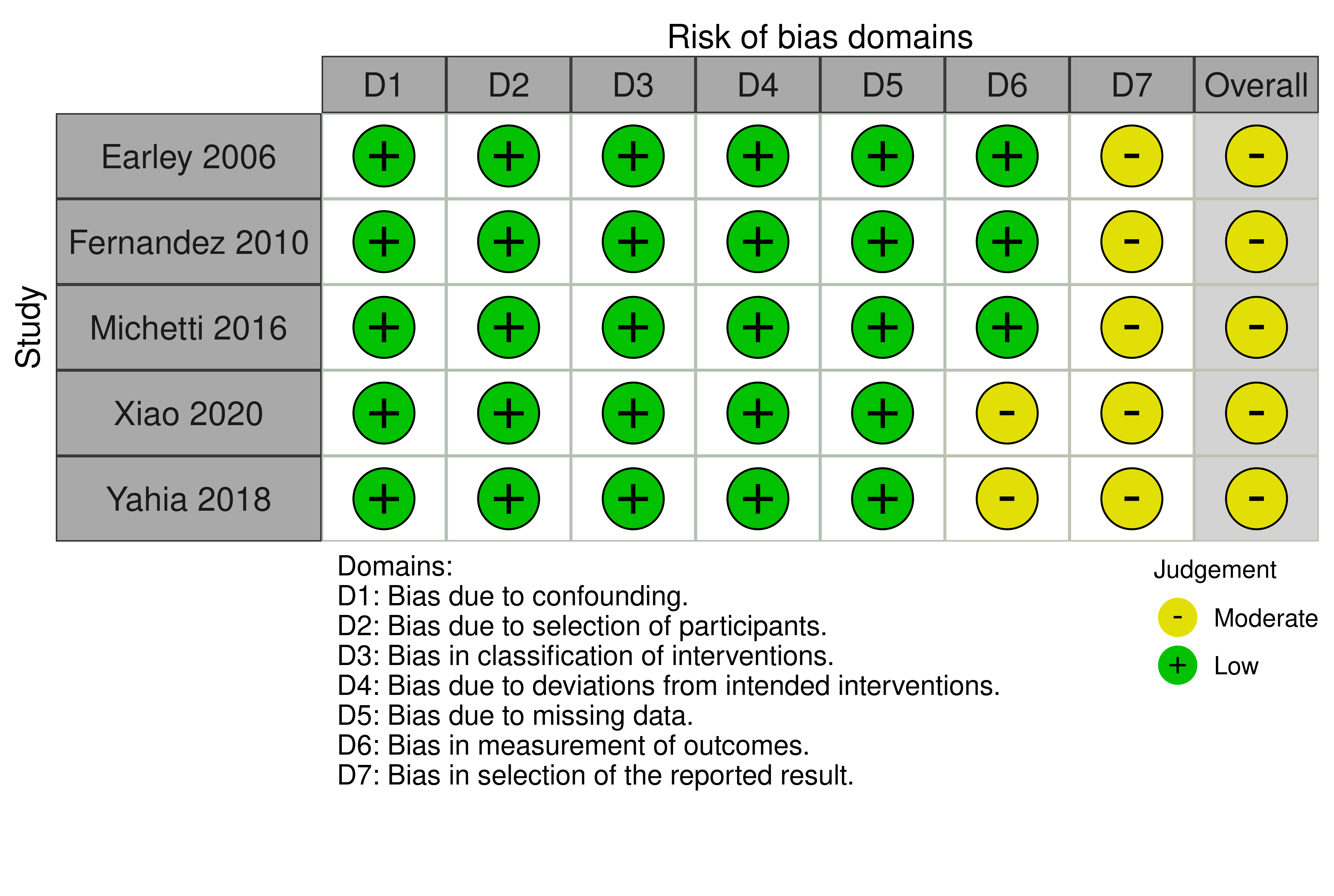


# **Figure S3. Bar plots for randomized controlled trials.**


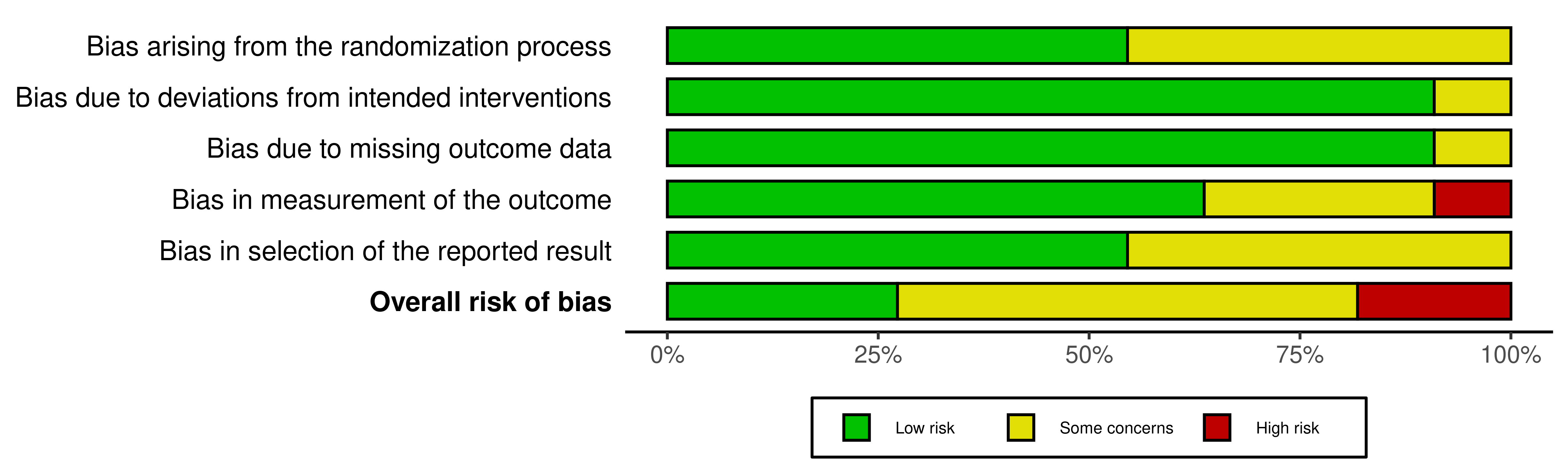


# **Figure S4. Bar plots for non-randomized controlled trials.**

We assessed the risk of bias by using ROVIS-I version 2 in non-randomized controlled trials, and domain 1 was rated as “low expect for concerns about uncontrolled confounding”.


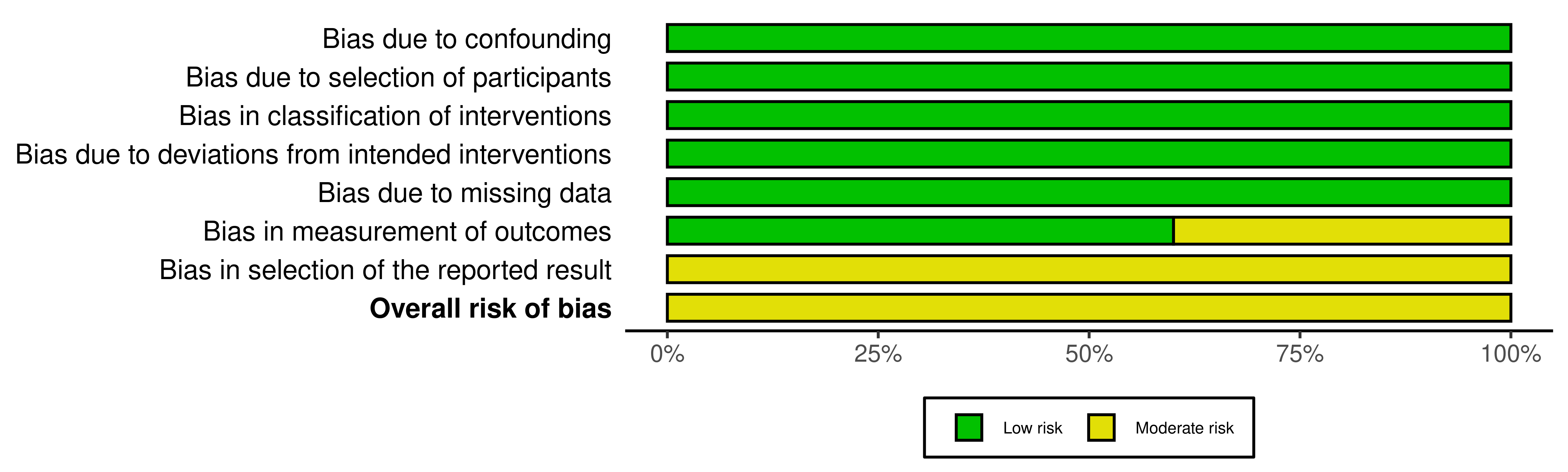


# **Figure S5. Sensitivity analysis excluding studies to assess the pharmacotherapy for anemia.**


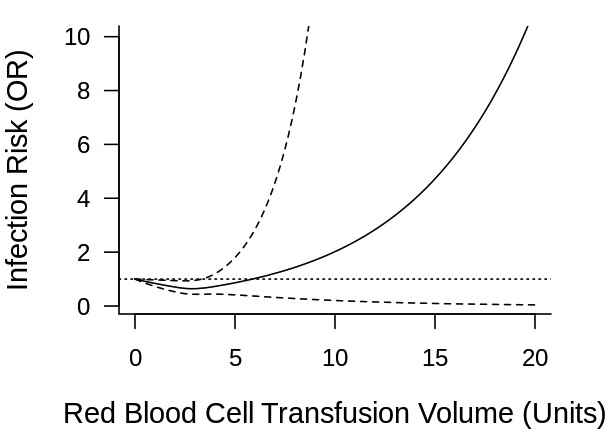


# **Figure S6. Sensitivity analysis excluding non-randomized controlled trials.**


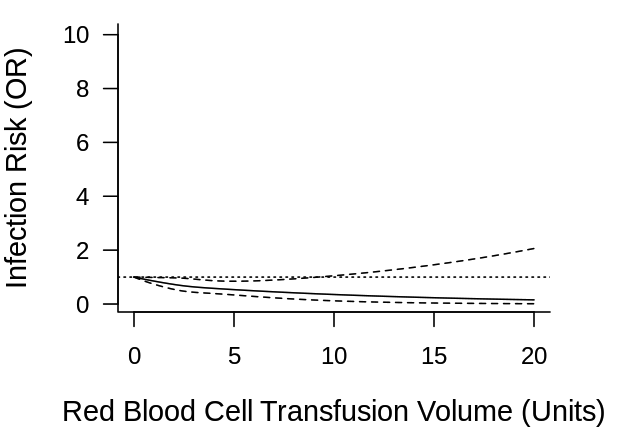


# **Figure S7. Sensitivity analysis for studies measuring ICU-acquired infections.**


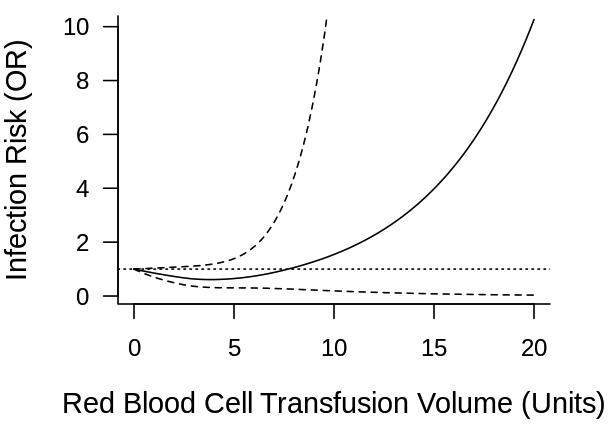


# **Figure S8. Sensitivity analysis for studies measuring Hospital-acquired infections.**

The data of hospital stay included the follow-up time over 28 days after randomization.


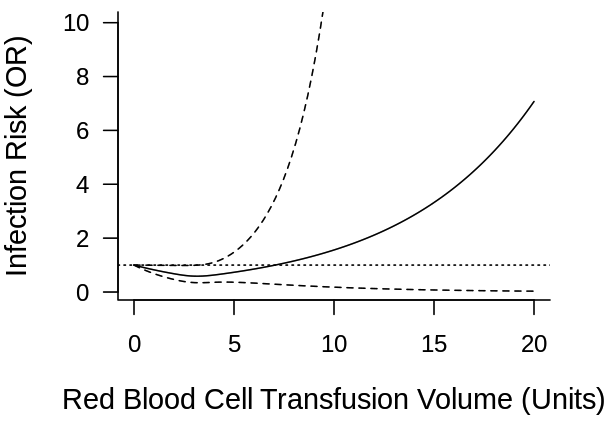


# **Figure S9. Sensitivity analysis for hospital-acquired infections in patients with sepsis/septic shock.**


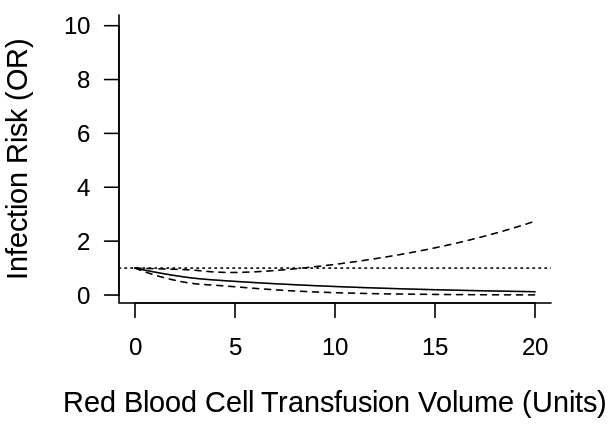


# **Figure S10. Sensitivity analysis for hospital-acquired infections in patients with bacteremia.**


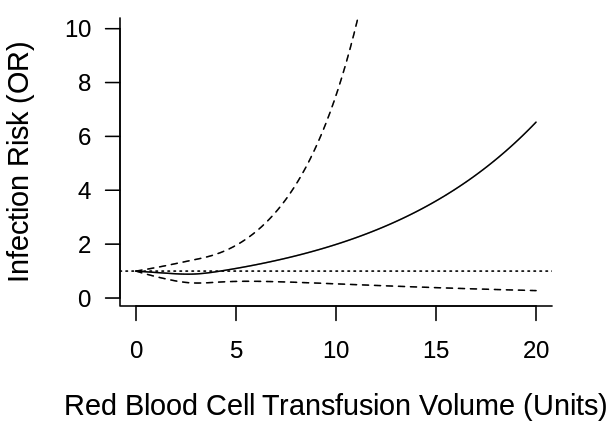


# **Figure S11. Sensitivity analysis for hospital-acquired infections in patients with any infections.**


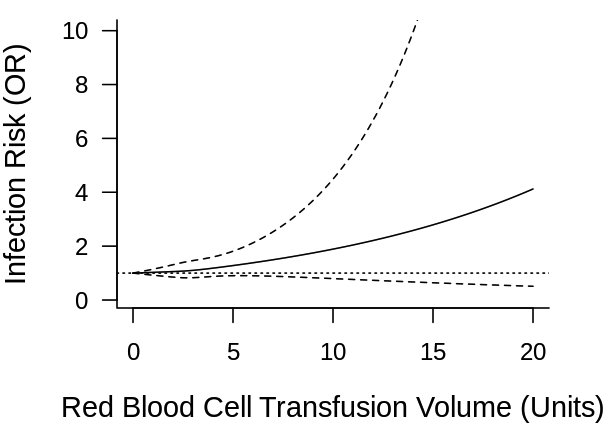


# **Figure S12. Sensitivity analysis for hospital-acquired infections in patients with pneumonia.**


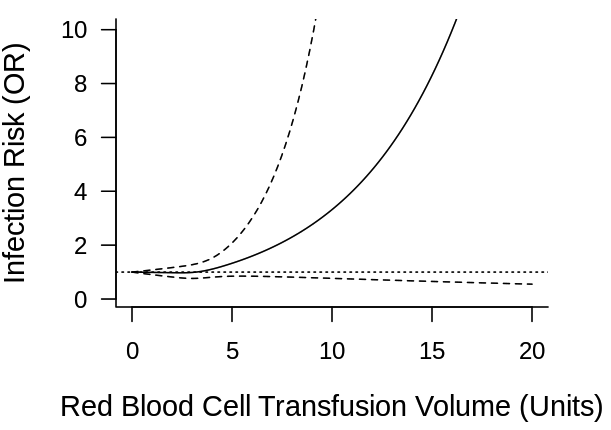


# **Figure S13. Sensitivity analysis for critically ill patients with trauma.**


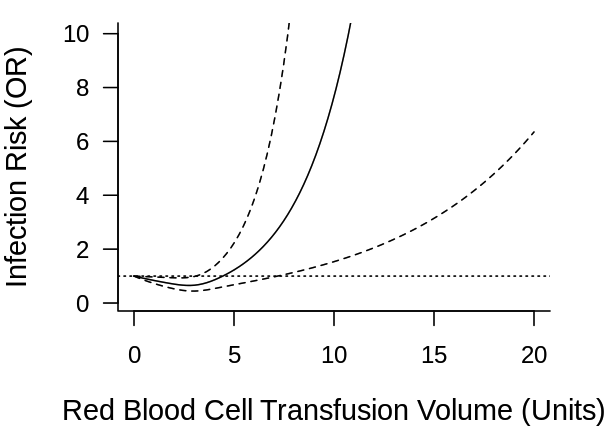


# **e-References**

1. Aubron C, Syres G, Nichol A, Bailey M, Board J, Magrin G, Murray L, Presneill J, Sutton J, Vallance S *et al*: A pilot feasibility trial of allocation of freshest available red blood cells versus standard care in critically ill patients. Transfusion 2012; 52(6):1196-202.

2. Bergamin FS, Almeida JP, Landoni G, Galas F, Fukushima JT, Fominskiy E, Park CHL, Osawa EA, Diz MPE, Oliveira GQ *et al*: Liberal Versus Restrictive Transfusion Strategy in Critically Ill Oncologic Patients: The Transfusion Requirements in Critically Ill Oncologic Patients Randomized Controlled Trial. Crit Care Med 2017; 45(5):766-73.

3. Fogagnolo A, Azzolina D, Taccone FS, Pedarzani E, Pasa G, Marianello D, Valpiani G, Marchesini C, Annoni F, Moureau A *et al*: Oxygen extraction-guided transfusion strategy in critically ill patients: study protocol for a randomised, open-labelled, controlled trial. BMJ Open 2024; 14(11):e089910.

4. Hébert PC, Wells G, Marshall J, Martin C, Tweeddale M, Pagliarello G, Blajchman M: Transfusion requirements in critical care. A pilot study. Canadian Critical Care Trials Group. Jama 1995; 273(18):1439-44.

5. Holst LB, Haase N, Wetterslev J, Wernerman J, Guttormsen AB, Karlsson S, Johansson PI, Aneman A, Vang ML, Winding R *et al*: Lower versus higher hemoglobin threshold for transfusion in septic shock. N Engl J Med 2014; 371(15):1381-91.

6. Juffermans NP, Prins DJ, Vlaar AP, Nieuwland R, Binnekade JM: Transfusion-related risk of secondary bacterial infections in sepsis patients: a retrospective cohort study. Shock 2011; 35(4):355-9.

7. Leal-Noval SR, Arellano-Orden V, Muñoz-Gómez M, Cayuela A, Marín-Caballos A, Rincón-Ferrari MD, García-Alfaro C, Amaya-Villar R, Casado-Méndez M, Dusseck R *et al*: Red Blood Cell Transfusion Guided by Near Infrared Spectroscopy in Neurocritically Ill Patients with Moderate or Severe Anemia: A Randomized, Controlled Trial. J Neurotrauma 2017; 34(17):2553-59.

8. . Transfusion in patients with onco-hematological malignancies resuscitated from septic shock (TRANSPORT). <https://clinicaltrials.gov/study/NCT03837171?cond=NCT03837171&rank=1>. Accessed 6 Jan 2025.

9. . Use of epoetin alfa and iron derisomaltose in treatment of anemia in patients with sepsis or septic shock: a randomized controlled trial (EpoAid). <https://clinicaltrials.gov/study/NCT06670963?cond=NCT06670963&rank=1>. Accessed 18 Jan 2025.

10. Nederpelt CJ, El Hechi M, Parks J, Fawley J, Mendoza AE, Saillant N, King DR, Fagenholz PJ, Velmahos GC, Kaafarani HMA: The dose-dependent relationship between blood transfusions and infections after trauma: A population-based study. J Trauma Acute Care Surg 2020; 89(1):51-57.

11. Walsh TS, McArdle F, McLellan SA, Maciver C, Maginnis M, Prescott RJ, McClelland DB: Does the storage time of transfused red blood cells influence regional or global indexes of tissue oxygenation in anemic critically ill patients? Crit Care Med 2004; 32(2):364-71.

12. Walsh TS, Boyd JA, Watson D, Hope D, Lewis S, Krishan A, Forbes JF, Ramsay P, Pearse R, Wallis C *et al*: Restrictive versus liberal transfusion strategies for older mechanically ventilated critically ill patients: a randomized pilot trial. Crit Care Med 2013; 41(10):2354-63.

13. Wang W, He Q, Zhu S, Wang M, Kang Y, Zhang R, Ji P, Zou K, Zong Z, Sun X: Association between blood transfusion and ventilator-associated events: a nested case-control study. Infect Control Hosp Epidemiol 2022; 43(5):597-602.

14. Zhang LM, Li R, Sun WB, Wang XP, Qi MM, Bai Y, Bai J, Zheng WC: Low-Dose, Early Fresh Frozen Plasma Transfusion Therapy After Severe Trauma Brain Injury: A Clinical, Prospective, Randomized, Controlled Study. World Neurosurg 2019; 132:e21-e27.

15. Bochicchio GV, Napolitano L, Joshi M, Bochicchio K, Shih D, Meyer W, Scalea TM: Blood product transfusion and ventilator-associated pneumonia in trauma patients. Surg Infect (Larchmt) 2008; 9(4):415-22.

16. Dupuis C, Garrouste-Orgeas M, Bailly S, Adrie C, Goldgran-Toledano D, Azoulay E, Ruckly S, Marcotte G, Souweine B, Darmon M *et al*: Effect of Transfusion on Mortality and Other Adverse Events Among Critically Ill Septic Patients: An Observational Study Using a Marginal Structural Cox Model. Crit Care Med 2017; 45(12):1972-80.

17. Engele LJ, Straat M, van Rooijen IHM, de Vooght KMK, Cremer OL, Schultz MJ, Bos LDJ, Juffermans NP, Consortium M: Transfusion of platelets, but not of red blood cells, is independently associated with nosocomial infections in the critically ill. Ann Intensive Care 2016; 6(1):67.

18. George ME, Skarda DE, Watts CR, Pham HD, Beilman GJ: Aggressive red blood cell transfusion: no association with improved outcomes for victims of isolated traumatic brain injury. Neurocrit Care 2008; 8(3):337-43.

19. Juffermans NP, Vlaar AP, Prins DJ, Goslings JC, Binnekade JM: The age of red blood cells is associated with bacterial infections in critically ill trauma patients. Blood Transfus 2012; 10(3):290-5.

20. Leal-Noval SR, Muñoz-Gómez M, Jiménez-Sánchez M, Cayuela A, Leal-Romero M, Puppo-Moreno A, Enamorado J, Arellano-Orden V: Red blood cell transfusion in non-bleeding critically ill patients with moderate anemia: is there a benefit? Intensive Care Med 2013; 39(3):445-53.

21. Michalia M, Kompoti M, Panagiotakopoulou A, Kallitsi G, Charitidi M, Trikka-Graphakos E, Clouva-Molyvdas PM: Impact of red blood cells transfusion on ICU-acquired bloodstream infections: a case-control study. J Crit Care 2012; 27(6):655-61.

22. Offner PJ, Moore EE, Biffl WL, Johnson JL, Silliman CC: Increased rate of infection associated with transfusion of old blood after severe injury. Arch Surg 2002; 137(6):711-6; discussion 16-7.

23. Péju E, Llitjos JF, Charpentier J, François A, Marin N, Cariou A, Chiche JD, Mira JP, Lambert J, Jamme M *et al*: Impact of Blood Product Transfusions on the Risk of ICU-Acquired Infections in Septic Shock. Crit Care Med 2021; 49(6):912-22.

24. Piriyapatsom A, Chaiwat O, Sak-Aroonchai J, Suwannasri W, Kanavitoon S: Incidence of red blood cell transfusion in mechanically ventilated surgical patients at Siriraj Hospital. J Med Assoc Thai 2014; 97(2):203-10.

25. Rachoin JS, Daher R, Schorr C, Milcarek B, Parrillo JE, Gerber DR: Microbiology, time course and clinical characteristics of infection in critically ill patients receiving packed red blood cell transfusion. Vox Sang 2009; 97(4):294-302.

26. Shorr AF, Jackson WL, Kelly KM, Fu M, Kollef MH: Transfusion practice and blood stream infections in critically ill patients. Chest 2005; 127(5):1722-8.

27. Taylor RW, Manganaro L, O'Brien J, Trottier SJ, Parkar N, Veremakis C: Impact of allogenic packed red blood cell transfusion on nosocomial infection rates in the critically ill patient. Crit Care Med 2002; 30(10):2249-54.

28. Taylor RW, O'Brien J, Trottier SJ, Manganaro L, Cytron M, Lesko MF, Arnzen K, Cappadoro C, Fu M, Plisco MS *et al*: Red blood cell transfusions and nosocomial infections in critically ill patients. Crit Care Med 2006; 34(9):2302-8; quiz 09.

29. Zilberberg MD, Stern LS, Wiederkehr DP, Doyle JJ, Shorr AF: Anemia, transfusions and hospital outcomes among critically ill patients on prolonged acute mechanical ventilation: a retrospective cohort study. Crit Care 2008; 12(2):R60.
